# Review of the genus *Genaemirum* Heinrich (Hymenoptera, Ichneumonidae, Ichneumoninae) with interactive identification keys to species

**DOI:** 10.3897/zookeys.636.10216

**Published:** 2016-11-24

**Authors:** Pascal Rousse, Gavin R. Broad, Simon van Noort

**Affiliations:** 1 Department of Natural History, Iziko Museums of South Africa, PO Box 61, Cape Town, 8000, South Africa; 2 Department of Life Sciences, the Natural History Museum, Cromwell Road, London SW75 BD, UK; 3 Department of Botany and Zoology, Evolutionary Genomics Group, Stellenbosch University, Private Bag X1, Stellenbosch 7602, South Africa; 4 Department of Biological Sciences, University of Cape Town, Private Bag, Rondebosch, 7701, South Africa

**Keywords:** Africa, Afrotropical region, biocontrol, Ichneumonidae, Ichneumoninae, host, identification key, parasitoid wasp, species description, systematics

## Abstract

We describe *Genaemirum phagocossorum* Rousse, Broad & van Noort, **sp. n.**, a new ichneumonine parasitoid wasp reared from *Eucalyptus nitens* logs infested by the cossid moth *Coryphodema tristis*, which is considered a major pest of forestry and food crops in South Africa. This is the first plausible host association for the genus, and fits with the host association predictions of Heinrich. Two further undescribed species were found in the collections of the Natural History Museum in London and are described as *Genaemirum phacochoerus* Broad, Rousse & van Noort, **sp. n.** and *Genaemirum fumosum* Broad, Rousse & van Noort, **sp. n.** An identification key to the eight known species and a diagnosis for each species are provided, including photographs of all the primary type specimens. Online Lucid interactive identification keys are available at: http://www.waspweb.org.

## Introduction

“To find the details of the life history of [*Genaemirum doryalidis*] and its host, would be one of the most rewarding tasks of the Ethiopian ichneumonology”, concluded Heinrich (1967) after his description of the “monstruous” (sic) projections he observed on the genae of *Genaemirum doryalidis* Heinrich. *Genaemirum* Heinrich, 1936 is an endemic Afrotropical genus with five species known to date. The genus is characterized by the flattened and uncarinate scutellum, the long and evenly curved propodeum with median areas fused into a long and smooth mid-longitudinal surface, combined with highly specialized head structures. Both sexes exhibit a more or less strong expansion of the junction of the occipital and hypostomal carinae, females have a strong transverse carina across the frons and some also have the lower genae more or less expanded backwards. Almost nothing is known about the biology of *Genaemirum* species. Heinrich (1967) hypothesized that the extraordinary head structure would be an adaptation to an unusual biology and noted that he would not be surprised to see them emerging from a dry wood borer, based on the fact that the holotype of *Genaemirum doryalidis* was recorded as having been reared from a tree. We recently obtained an undescribed species of the genus, reared from a log infested with wood-boring moth larvae suggesting that Heinrich was in all likelihood right. We also take the opportunity to describe additional species that lay undescribed in the BMNH collection, provide an identification key to the eight known species, including online Lucid keys available at www.waspweb.org, and present high resolution images of all the primary type specimens.

## Material and methods

### Depositories

BMNH
 Natural History Museum, London, UK (Gavin Broad & David Notton) 


MNHN
 Muséum National d’Histoire Naturelle, Paris, France (Claire Villemant & Agnièle Touret-Alby) 


SAMC
 Iziko South African Museum, Cape Town, South Africa (Simon van Noort) 


ZMHB
 Museum für Naturkunde, Humboldt Universität, Berlin, Germany (Frank Koch & Viola Richter) 


ZSMC
 Zoologische Staatsammlung, München, Germany (Stefan Schmidt) 


### Photographs

At SAMC we used a Leica LAS 4.4 imaging system, which comprised a Leica® Z16 microscope with a Leica DFC450 Camera with 0.63× video objective attached. The imaging process, using an automated Z-stepper, was managed using the Leica Application Suite V 4.4 software installed on a desktop computer. At BMNH, images were acquired using a Canon SLR EOS 5DSR with 65 mm macro lens mounted on a copy stand with an automated Z-stepper; images were aligned using Helicon Focus software version 6.6.1. Diffused lighting was achieved using techniques summarized in Buffington et al. (2005), Kerr et al. (2008), and Buffington and Gates (2009). All images presented in this paper, as well as supplementary images, are available at www.waspweb.org.

## Results

### Genaemirum

Taxon classificationAnimaliaHymenopteraIchneumonidae

Heinrich, 1936

#### Diagnosis

(updated after Heinrich 1936, 1967). *Female.* Flagellum filiform, medium-sized and stout, not distinctly flattened and not to slightly widened beyond middle; head thick, temple long, wide and curved behind eyes; malar space very short, usually distinctly shorter than mandibular base; lower gena sometimes expanded backwards into more or less projecting protrusions; frons crossed by a laterally sinuate to acutely pointed transverse carina; oral and hypostomal carinae junction produced into a more or less strong triangular protuberance; mandible rather stout, upper tooth slightly to significantly longer than lower tooth; ventral margin of clypeus truncate to variably produced; mesoscutum moderately rounded with notaulus distinct on anterior third; scutellum flat to weakly convex, without lateral carina; propodeum long, in profile evenly and gently curved without distinct separation between anterior and posterior part, lateral areas curved down almost to base of hind coxa, median areas amalgamated into one elongate mid-longitudinal area which is not separated from post-scutellum; legs rather stout, hind coxa without scopa; fore wing with cu–a distal to Rs&M, areolet pentagonal and strongly narrowed anteriorly; first tergite with a distinct median field; second tergite and base of following tergites usually longitudinally sculptured; gastrocoelus deep, large; metasoma strongly oxypygous, apical margin of hypopygium remote from base of ovipositor sheath. *Male* (known for two species only). Sexual dimorphism very limited: flagellum more slender with tyloids, lower gena without protrusion, frons with transverse carina absent or very weak, pale markings more extensive.

#### Genotype.

*Genaemirum mesoleucum* Heinrich, 1936.

#### Differential diagnosis.

The gradual curve of the propodeum in profile is typical of the tribe Heresiarchini. The longitudinal confluence of the median propodeal areas, combined with the weakness of the basal furrow, is characteristic of several Afrotropical genera. *Genaemirum* is similar to *Coelichneumon* Thomson, 1893, absent from the Afrotropical region, in which the notauli are indistinct and the propodeum is shorter with areas basalis and superomedia separated. *Genaemirum* is also morphologically similar to the Afrotropical genus *Afrocoelichneumon* Heinrich, 1938, in which the mid-longitudinal area of the propodeum is wider and the specialized structures of the female totally absent.

#### Biology.

Heinrich (1967) did not speculate on exactly how the remarkable adaptations of the head could be associated with "some extraordinary biological features", but presumably the head is adapted to access the host in some unique way or is adapted for emerging from a particular substrate. Based on the oxypygous metasoma (the hypopygium is relatively short and ovipositor relatively long) and the biology of *Coelichneumon* species, *Genaemirum* species are likely to be idiobiont parasitoids of pupae. Many of the Carpenter moths (Cossidae), including the probable host of *Paropta phagocossorum*, pupate inside tunnels in wood (Gebeyehu et al. 2005, Plaut 1973), hence the head specializations exhibited by *Genaemirum* species are predicted to be an adaptation assisting the females to crawl down the galleries when searching for their host. It would be very interesting to see what the males look like in the species with very extravagant head ornamentation as this could shed light on the possible functional significance of the protuberances. However, in the two species where males are known (*Genaemirum varianum* and *Genaemirum phagocossorum*) the male head does not exhibit any morphological adaptations, suggesting that the underlying evolutionary drivers are acting on the females only. Selection for development of these facial protrusions would then stem from an increased functional ability to find hosts for oviposition, and are likely to have evolved to facilitate forward progress through the host caterpillars’ frass that blocks their feeding tunnels. The females would need to negotiate these extended physical hurdles to reach the host pupae. The facial protrusions are very spade or blade-like in their form and we hypothesize that these protrusions act in a mechanical fashion, forcing the frass to open up, much like a road grader with an angled blade that pushes soil to the side.

Interestingly, Tom Huddleston’s note in the BMNH copy of Heinrich’s monograph indicates that some *Genaemirum* sp. individuals, identified by J.F. Perkins, were purportedly associated with *Eulophonotus myrmeleon* Felder (Lepidoptera: Cossidae). This ichneumonid genus is thus apparently associated with the cossid moth family. These specimens appear not to have been retained at BMNH and their identification is unknown, although Perkins apparently noted that these represented a species not included by Heinrich (1967).

### Key to species (updated after Heinrich 1967)

Online Lucid identification keys are available at: www.waspweb.org

**Table:** 

	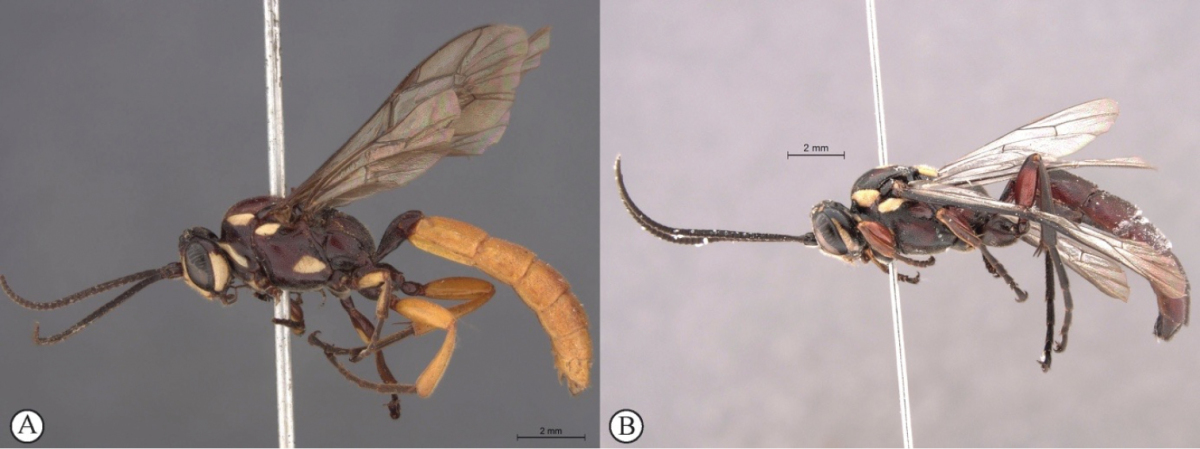	
1	Male: known only for *Genaemirum varianum* (A) and *Genaemirum phagocossorum* (B)	**2**
	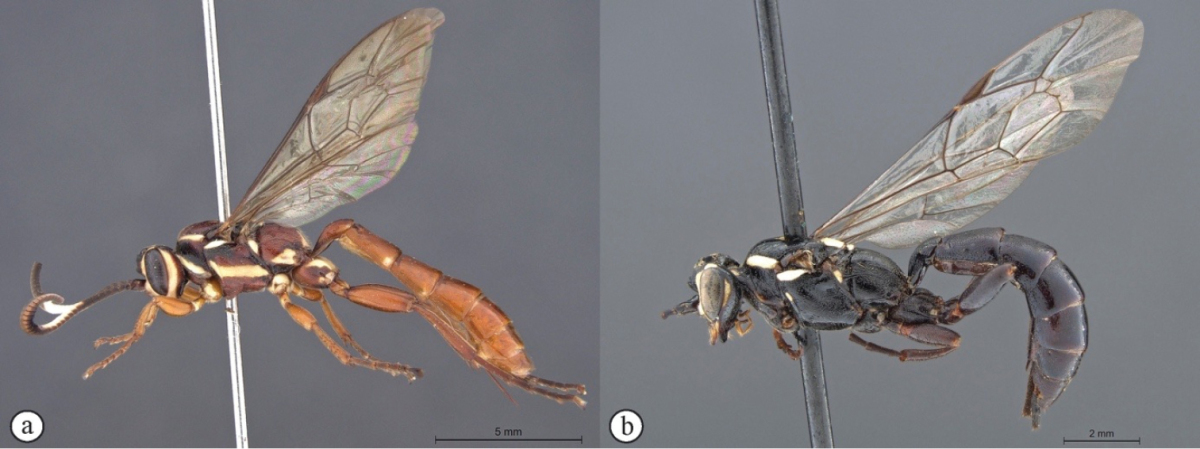	
–	Female (a, b)	**3**
	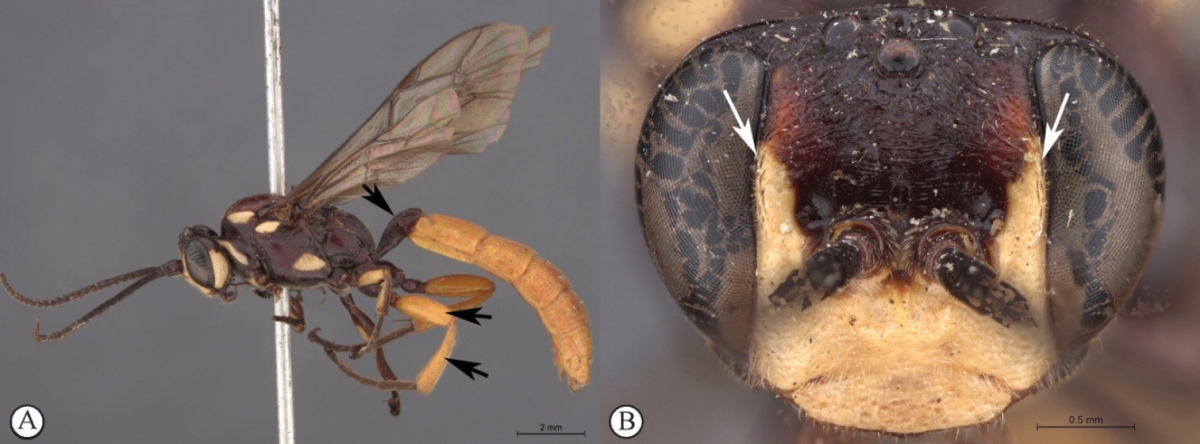	
2	Metasoma yellowish-orange with tergite 1 dark reddish-brown (A); hind femur and tibia yellowish-orange (A); antennal scrobe extends to eye margins (B)	***Genaemirum varianum* (Tosquinet)**
	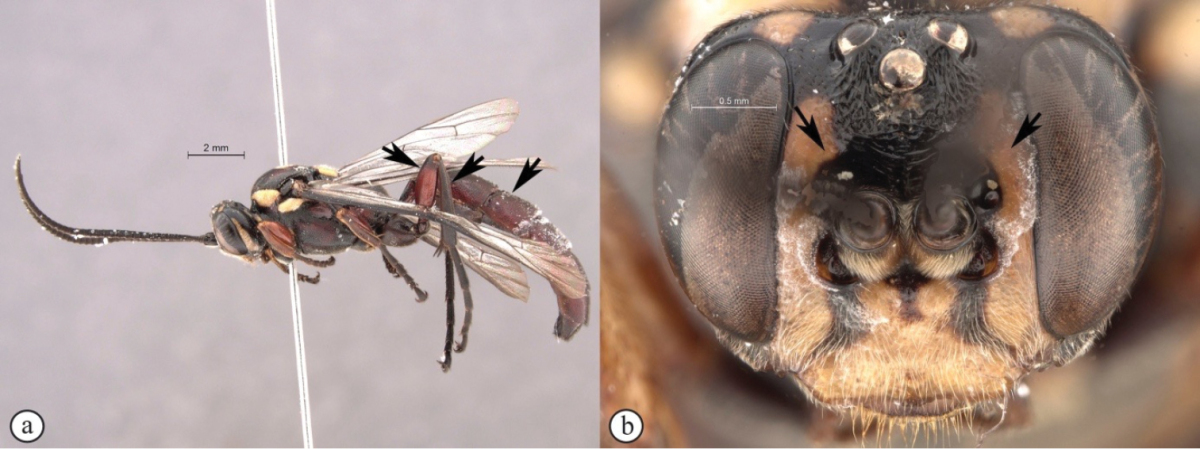	
–	Metasoma uniformly dark reddish-brown (a); hind femur and tibia dark reddish-brown (a); antennal scrobe less extensive, ending an ocellar diameter from eye margins (b)	***Genaemirum phagocossorum* Rousse, Broad & van Noort, sp. n.**
	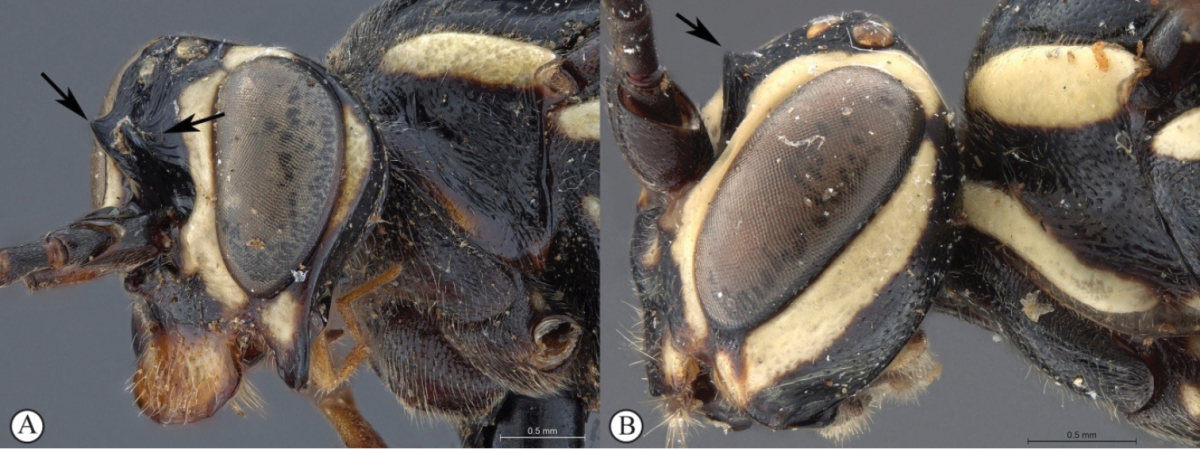	
3	Frons with pronounced, horn-like projections pointed upward (A, B)	**4**
	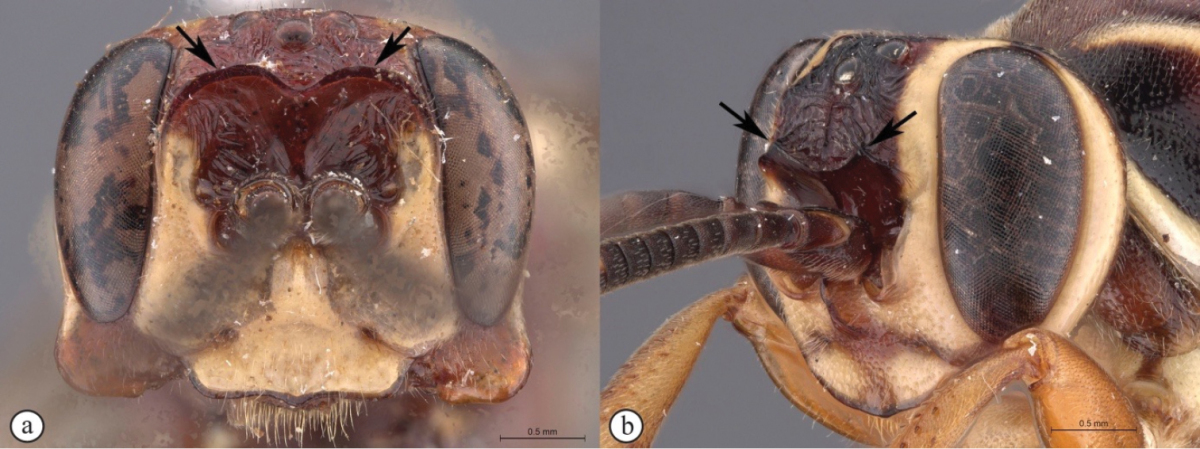	
–	Frons with a bisinuate transverse lamella (a), that may have minor projections (b)	**7**
	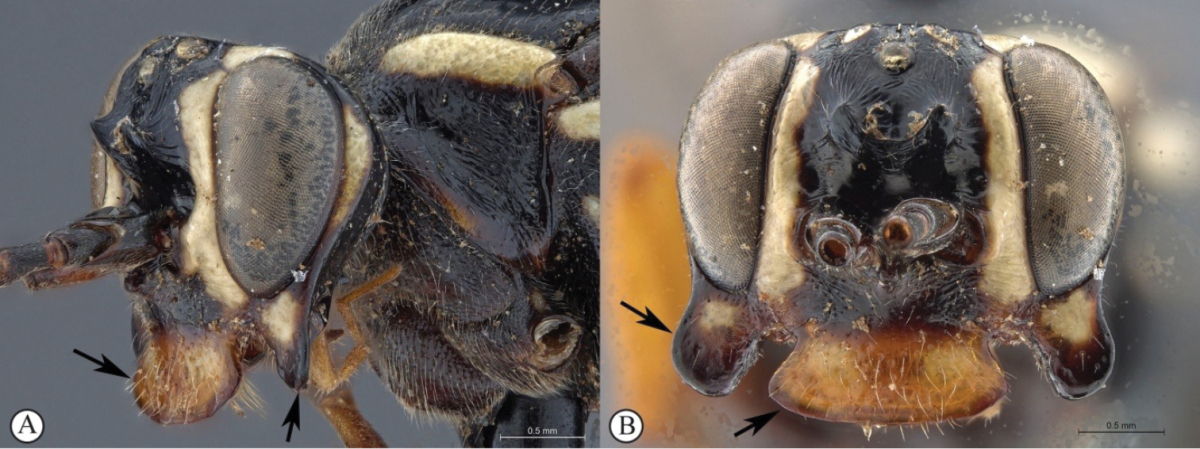	
4	Lower gena produced into a long spade-like protrusion (A, B); clypeus exceptionally expanded (A, B)	**5**
	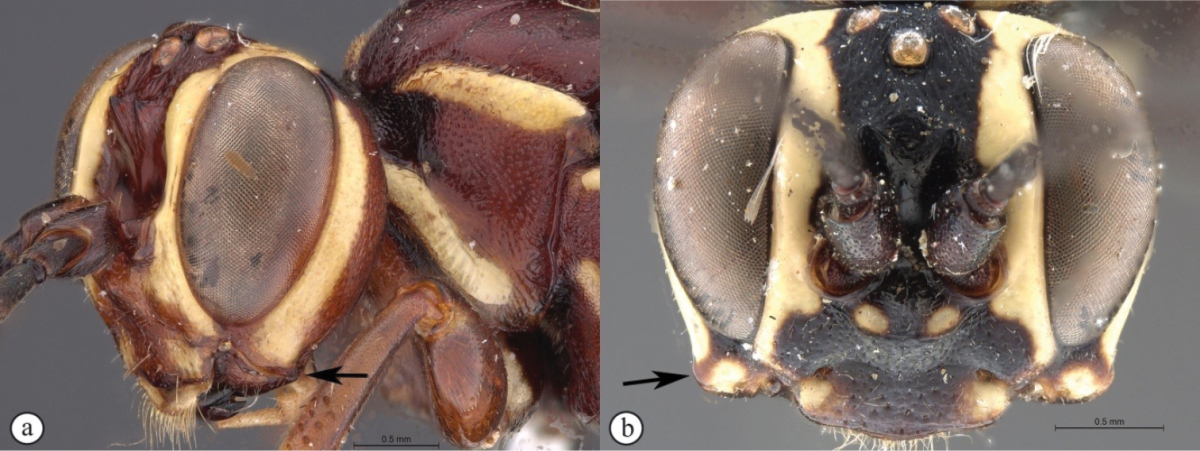	
–	Lower gena not expanded (a), or distinctly less expanded into a backward curved collar (b)	**6**
	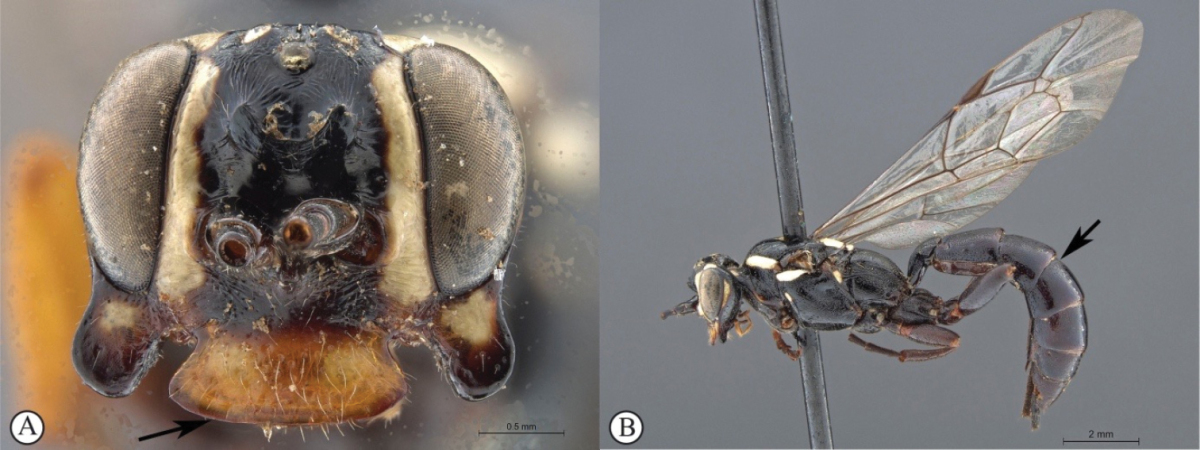	
5	Clypeus apically slightly convex, gena moderately protruded (A); metasoma brownish-black (B)	***Genaemirum doryalidis* Heinrich**
	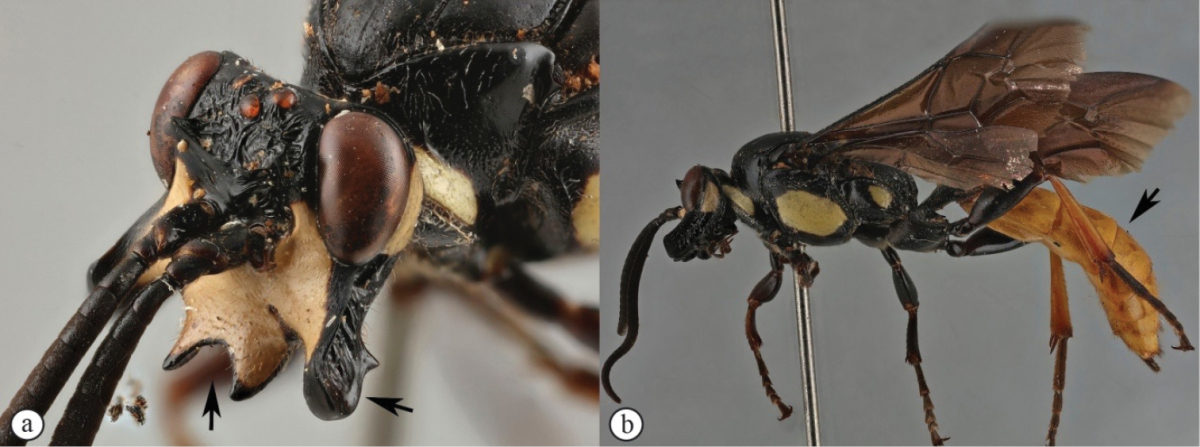	
–	Clypeus apically deeply emarginate, gena dramatically protruded (a); metasoma yellowish-orange (b)	***Genaemirum phacochoerus* Broad, Rousse & van Noort, sp. n.**
	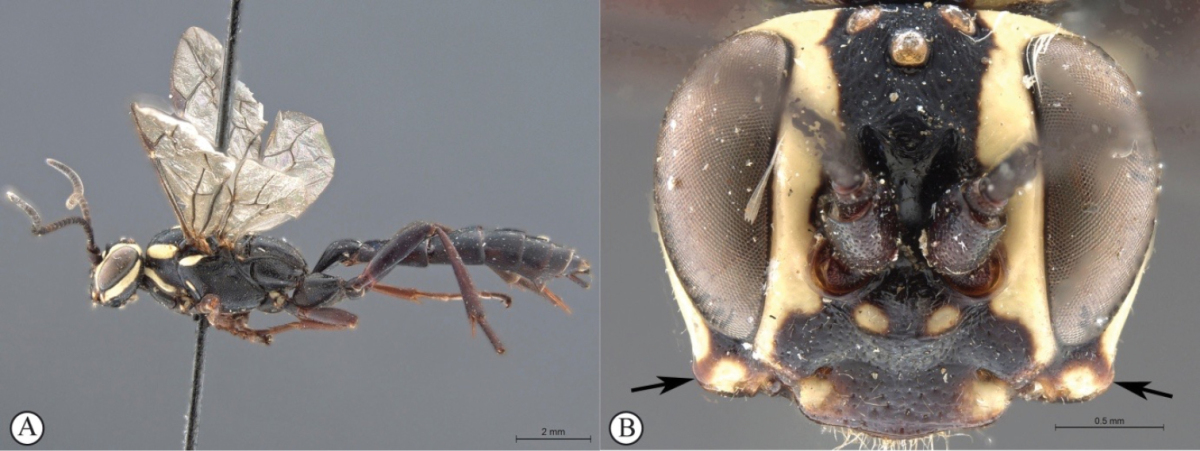	
6	General coloration black (A); lower gena expanded into a backward curved collar (B)	***Genaemirum mesoleucum* Heinrich**
	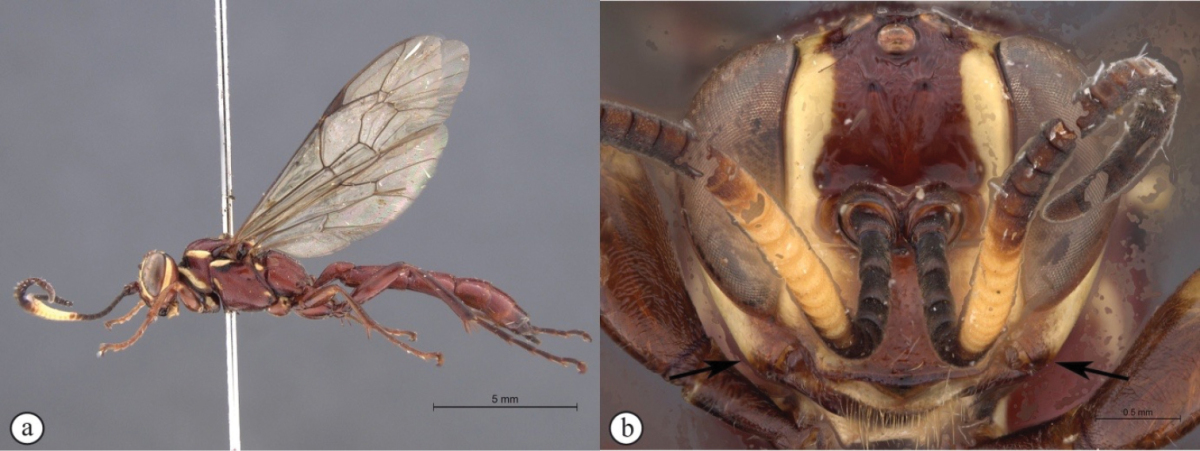	
–	General coloration dark red (a); lower gena not expanded (b)	***Genaemirum vulcanicola* Heinrich**
	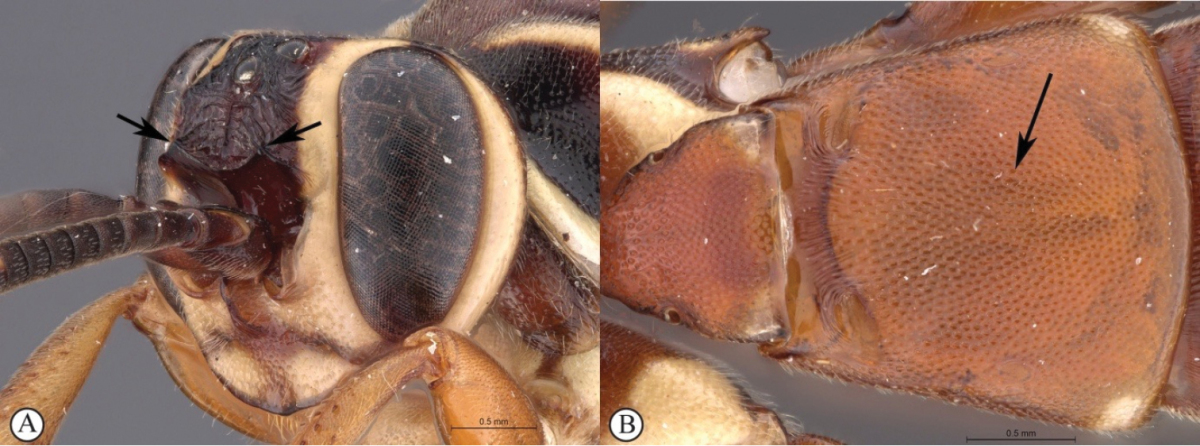	
7	Frons with a forked, horn-like projecting carina above the antennal scrobes (A); second tergite medially punctate (B)	***Genaemirum rhinoceros* Heinrich**
	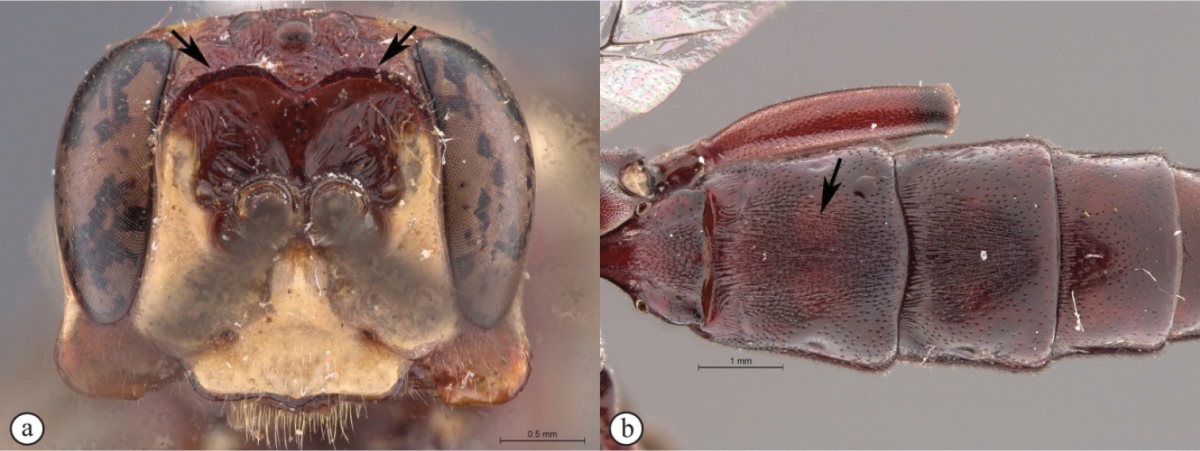	
–	Frons with a broad to acute cordate lamella above the antennal scrobes (a); second tergite medially longitudinally strigose (metasoma lacking in ♀ allotype of *Genaemirum varianum*, but ♂ holotype tergite is longitudinally strigose) (b)	**8**
	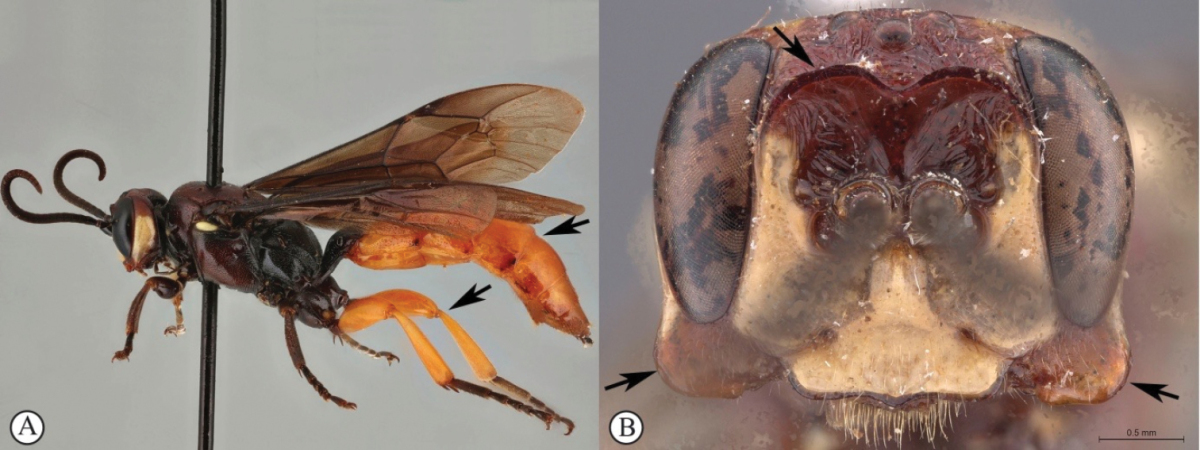	
8	Metasoma, hind femora and tibiae yellowish-orange (A); frons with a broad cordate lamella above the antennal scrobes (B)	**9**
	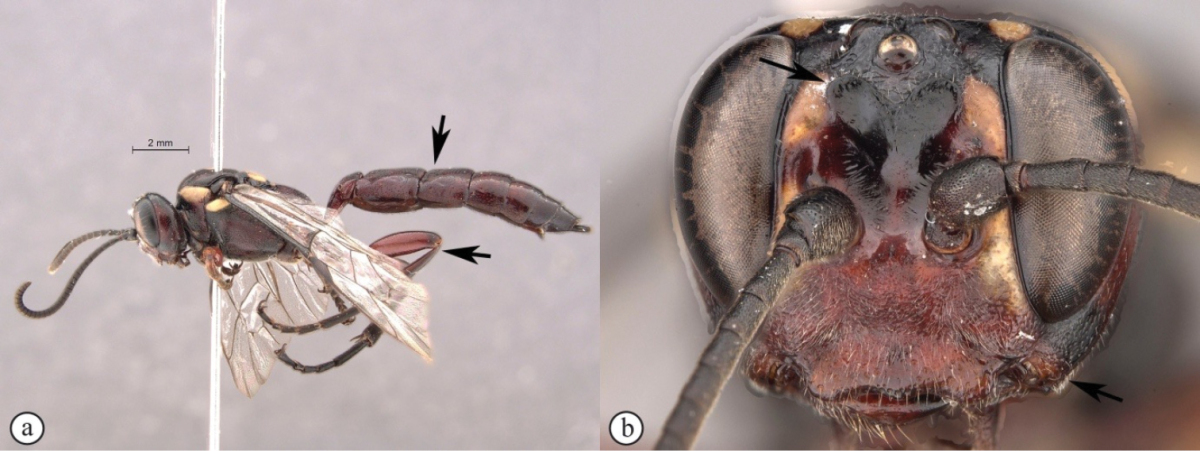	
–	Metasoma, hind femora and tibiae reddish (a); frons with an acute cordate lamella above the antennal scrobes (b)	***Genaemirum phagocossorum* Rousse, Broad & van Noort, sp. n.**
	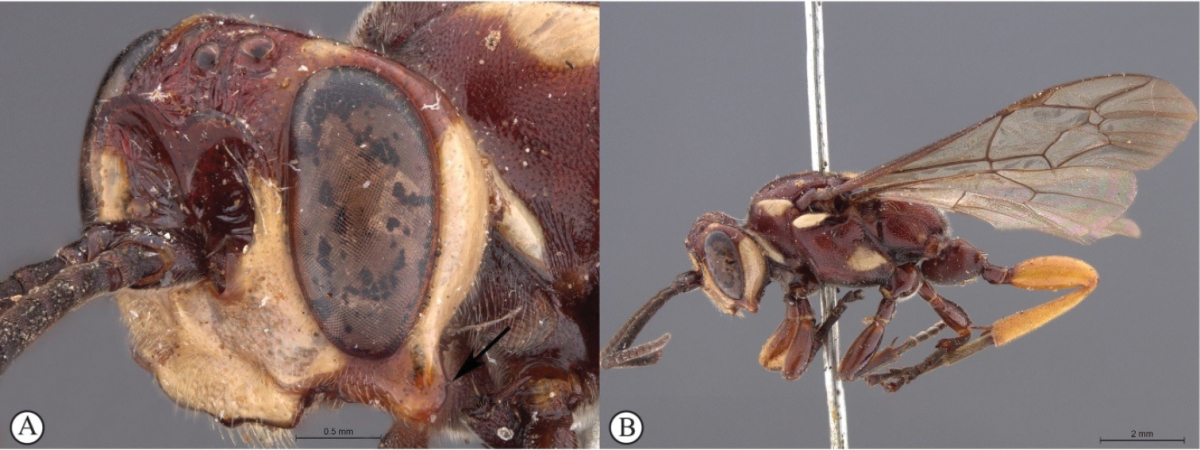	
9	Lower gena expanded into a backward curved, bluntly triangular, protuberance (A); wings hyaline (B)	***Genaemirum varianum* (Tosquinet)**
	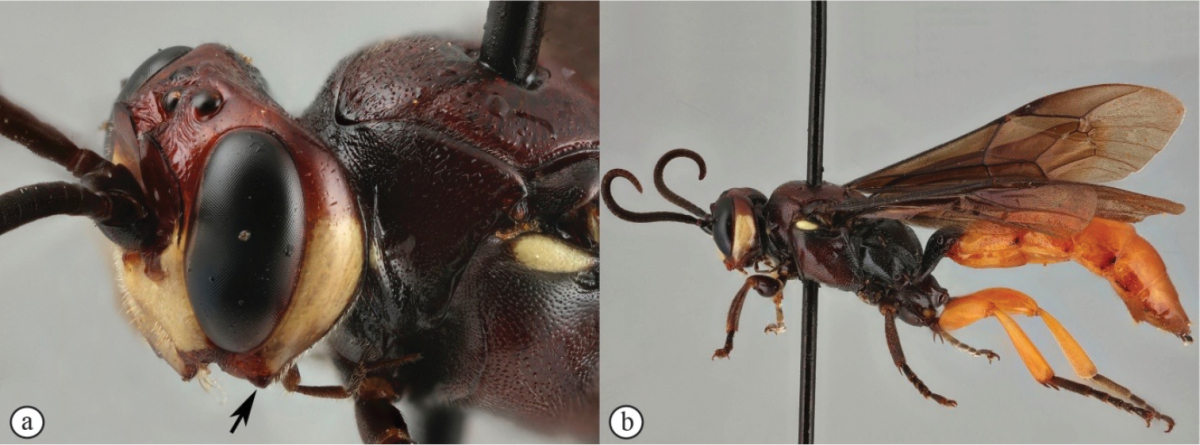	
–	Lower gena not expanded into a backward curved protuberance (a); wings infuscate (b)	***Genaemirum fumosum* Broad, Rousse & van Noort, sp. n.**

### Descriptions and diagnoses

#### Genaemirum phagocossorum

Taxon classificationAnimaliaHymenopteraIchneumonidae

Rousse, Broad & van Noort sp. n.

http://zoobank.org/64E4668F-C774-40B8-8D66-2FA090523F07

Figs 1 , 2

##### Type material.

**HOLOTYPE** ♀: SOUTH AFRICA, Mpumalanga, Sappi Ndubazi plantation, near Machadadorp, N. 2006, B. Slippers, emerged from *Eucalyptus nitens* logs infested with cossid larvae of *Coryphodema tristis*, SAM–HYM–P025037a (SAMC). **PARATYPE.** 1♂ same label data, SAM–HYM–P025037b (SAMC).

##### Diagnosis.

*Female.* Body length 16 mm. Black to dark reddish-brown overall with isolated yellow markings on head and mesosoma (pronotal shoulders, middle of mesoscutum, scutellum, dorsal section of mesopleuron below tegulae); vertex black with two small isolated lateral yellow spots, scrobe black dorsally and medially, dark reddish-brown laterally, separated from inner eye margins by yellow horizontal bands; lower face reddish-brown; legs black to dark reddish-brown; wings hyaline; antenna with 30 flagellomeres; lower gena not produced laterally; clypeus transverse, with ventral margin trisinuate; lower face reticulate, separated from upper face by a transverse ledge below toruli; upper frons and vertex smooth, with localised weak striations in an otherwise polished scrobe; scrobe dorsally demarcated by cordate raised sublamelliform carina; mesosoma sparsely punctate; metasoma sparsely punctate, polished, except for tergites 1-3, which are medially striate. *Male.* Body length 16 mm. Colouration similar to female except for face, which is dirty yellow with two black bands extending from toruli to clypeal-genal junction; vertex black with lateral yellow spots; gena with vertical yellow band adjacent to posterior eye margin; propodeum dorsally black; lower gena normal and frons without transverse carina; scrobe with horizontally curved striations; ocellar triangle reticulate, black; metasomal tergites more densely punctate than in female.

##### Differential diagnosis.

The uniquely shaped cordate, sublamelliform raised carina dorsally demarcating the scrobe readily separates females of this new species from all the other described species, each of which has a diagnostically shaped horizontal carina, with various uniquely shaped projections in this region. The male is distinguishable from the only other known male (*Genaemirum varianum*) by the extent of the scrobe: extending to the inner eye margins in *Genaemirum varianum*, terminating laterally well before the inner eye margin in *Genaemirum phagocossorum*; scrobe sculpture, which is less extensive in *Genaemirum phagocossorum*; and overall body colour (body uniformly dark reddish-brown in *Genaemirum phagocossorum*; metasoma and hind femora and tibiae ochreous yellow, contrasting with dark reddish-brown head and mesosoma in *Genaemirum varianum*).

##### Etymology.

From the latin “cossus” for “worm or grub found in wood”, which is the likely host (Cossidae), and “phago” = latin for “a glutton”. Noun in the genitive case.

##### Description.

FEMALE. *Color.* Head dark reddish fading to black dorsally and on lower gena, with yellow markings: facial and frontal orbits and a postero-lateral isolated dot on vertex; mesosoma very dark reddish fading to black dorsally, with yellow markings: dorsal margin of pronotum, subtegular ridge, centre of mesoscutum, scutellum and middle of metanotum; metasoma uniformly dark reddish; flagellum uniformly black; legs dark reddish, mid and hind tibiae and tarsi black; wings hyaline, venation testaceous to black.

*Head.* Mandible rather stout, mid-longitudinally densely punctate, teeth smoother with upper tooth about twice as long as lower tooth; malar space 0.5× as long as mandible basal width; lower gena not expanded; occipital and hypostomal carinae joining at 0.6× basal mandible width before mandible, distinctly expanded at their junction into a blunt triangle; clypeus strongly transverse, about twice as wide as high, in profile flat, ventral margin with median and lateral lobes, unevenly punctate with punctation denser dorsally; face strongly transverse, about three times as wide as high, laterally punctate on coriaceous background, punctation denser medially on transversely rugose background; lower frons quite smooth, flat and steeply elevated above toruli, separated from upper frons by a bisinuate transverse carina; upper frons coarsely rugose; vertex coriaceous with sparse punctures, punctation distinctly denser on inter-ocellar area; temple and gena densely punctate; temple moderately swollen behind eye; ocellar triangle wider than long, ocelli rather small, POL 1.0, OOL 1.3; antenna stout, widened slightly before middle, with 30 flagellomeres, basally elongate, flagellomeres 7–9 subquadrate, following flagellomeres transverse.

*Mesosoma.* Pronotum finely and densely punctate fading ventrally to coarsely longitudinally puncto-striate, pronotum collar enlarged and epomia strong; mesopleuron finely and densely punctate, somewhat rugose-punctate postero-ventrally, speculum barely smoother ventrally, posterior suture deeply crenulate, epicnemial carina nearly reaching pronotal margin below subtegular ridge, subtegular ridge strongly expanded; metapleuron densely and finely punctate, crenulate along pleural carina; mesoscutum evenly rounded, finely and densely punctate, notaulus distinct to anterior third, scuto-scutellar groove quite smooth; scutellum finely and more sparsely punctate, without lateral carina, quite flat in profile; propodeum typical of the genus, gently and evenly rounded in profile with median areas fused, carination distinct but very weak, densely punctate with punctation obsolescent mid-basally and distinctly coarser mid-posteriorly. *Legs.* Fore and mid tibiae with short and rather dense bristles along anterior faces, bristle sockets large and cupular.

*Metasoma.* Petiole with lateral and submedian dorsal carinae strong, abruptly enlarged into second tergite from level of spiracle; second tergite sparsely punctate laterally, medially longitudinally strigose; following tergites unevenly and sparsely punctate except tergite 2 medially and tergite 3 mid-basally longitudinally strigose; gastrocoelus deep, thyridia separated by 1.6× their own width; ovipositor sheath slightly protruding beyond metasomal apex.

**Figure 1.**
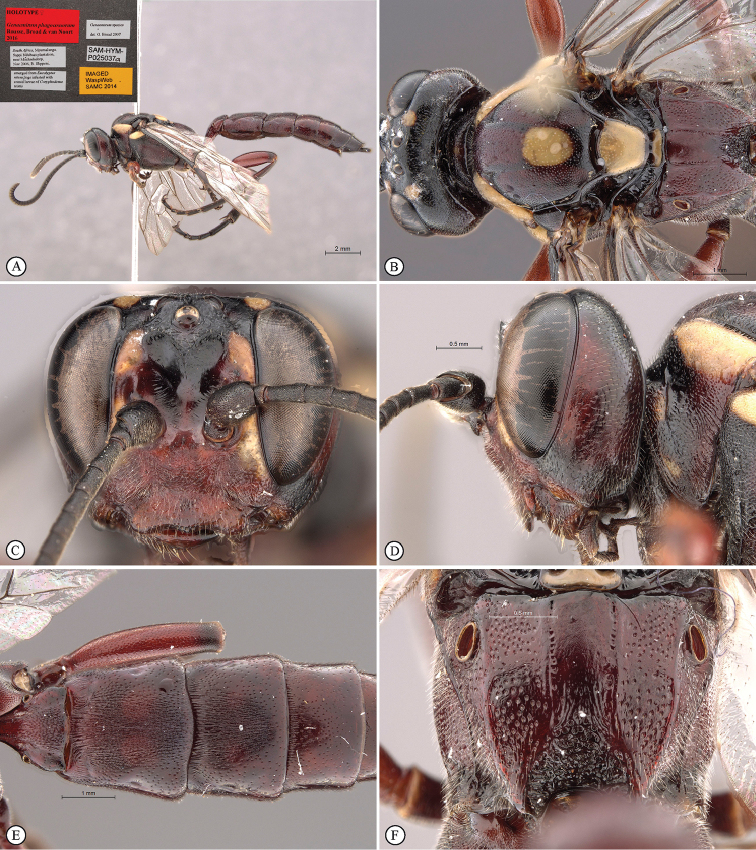
*Genaemirum phagocossorum* sp. n. Holotype female. **A** habitus lateral view (inset: data labels) **B** head, mesosoma, dorsal view **C** head anterior view **D** head, mesosoma anterior-lateral view **E** metasomal tergites 1-4 dorsal view **F** propodeum, dorsal view.

MALE. Yellow markings more expanded, encompassing also mandible, clypeus, median face, genal orbits and pronotal collar; flagellum not widened with 33 flagellomeres, flagellomeres 7–14 with tyloids on outer surface and 6–32 with differentiated bristle mid-ventrally; lower frons transversely striate, transverse carina strongly attenuated; parameres simple; otherwise similar to female.

**Figure 2.**
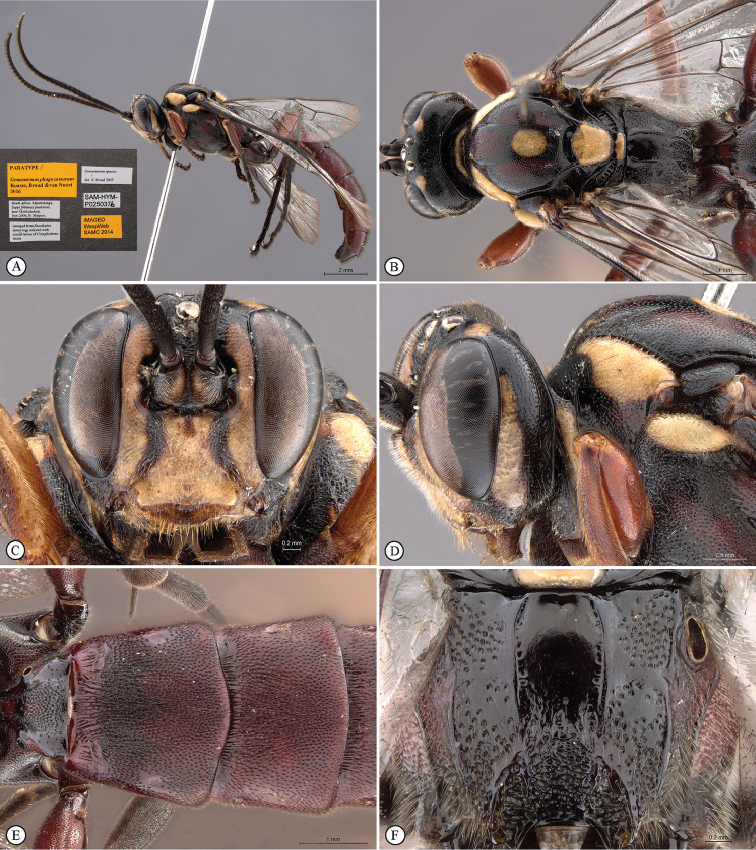
*Genaemirum phagocossorum* sp. n. Paratype male. **A** habitus lateral view (inset: data labels) **B** head, mesosoma, dorsal view **C** head anterior view **D** head, mesosoma anterior-lateral view **E** metasomal tergites 1-4 dorsal view **F** propodeum, dorsal view.

##### Biology.

Reared from logs of *Eucalyptus nitens* (H. Deane & Maiden) Maiden (Myrtales: Myrtaceae), which were heavily infested with *Coryphodema tristis* (Drury) (Lepidoptera: Cossidae), but specific host association was not established. *Coryphodema tristis* is regarded as an agricultural pest in southern Africa because its host-plant range includes food crops, chiefly quince and apple trees (Rosales: Rosaceae, *Cydonia oblonga* Miller and *Malus pumila* Miller var. *domestica* Schneider). It is also considered to be a major forestry pest of *Eucalyptus nitens*, which is extensively cultivated in South Africa for the pulp and mining industry (Gebeyehu et al. 2005, Boreham 2006, Adam et al. 2013, Degefu et al. 2013). This likely host record suggests the potential for using *Genaemirum phagocossorum* sp. n. as a biocontrol agent of this pest species. However, the low number of reared specimens suggests that it is not a common parasitoid of this lepidopteran pest. See further discussion on biology under the genus treatment.

##### Distribution.

South Africa.

#### Genaemirum phacochoerus

Taxon classificationAnimaliaHymenopteraIchneumonidae

Broad, Rousse & van Noort sp. n.

http://zoobank.org/3A7FE578-617F-4ED3-B9EC-C4778D202B25

Fig. 3

##### Type material.

**HOLOTYPE** ♀: [TANZANIA] ‘Mahali Peninsula. I 10.ix.1959. 2^nd^. Oxford U[niversity]. Exped[ition]. B.M. 1960-279.’ ‘Kungwe Camp: Forest clearing. 6’000ft.’ (BMNH).

##### Diagnosis.

*Female.* Body length 16 mm, fore wing length 13mm. Black mesosoma with large creamy-white markings on head and mesosoma and metasoma dull yellow beyond black first segment; face, large spot on upper vertex, lower edge of pronotum, scutellum, large oval patch on mesopleuron and spot on metapleuron all creamy-white; legs black except for dull reddish hind tibia; wings strongly infumate; antenna with 29 flagellomeres; lower gena produced laterally as a massive, rounded protuberance, concave and heavily rugose on outer side, with a triangular projection on its lower edge at half length; hypostomal carina produced as large, rounded triangular projection; clypeus slightly concave, corners elongate with edge deeply emarginate; lower face coriaceous, slightly concave, with pointed projection between antennal sockets; upper frons rugose; scrobe smooth, impunctate, with some weak rugae; scrobe dorsally demarcated by triangularly pointed projections; mesosoma mostly coriaceous, sparsely punctate; metasoma with sparse but distinct punctures, coriaceous, except tergites 2-3 medially strigose. *Male.* Unknown.

**Figure 3.**
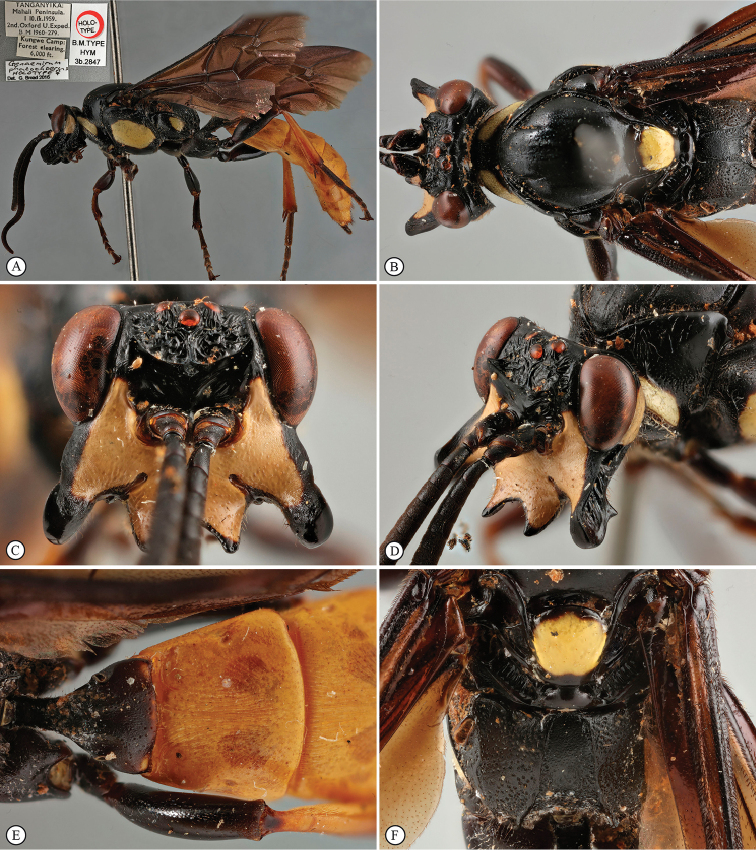
*Genaemirum phacochoerus* sp. n. Holotype female. **A** habitus lateral view (inset: data labels) **B** head, mesosoma, dorsal view **C** head anterior view **D** head, mesosoma anterior-lateral view **E** metasomal tergites 1-4 dorsal view **F** propodeum, dorsal view.

##### Differential diagnosis.

The unique clypeus, which is deeply emarginate with long, sharp corners, and the exceedingly long, strongly sculptured genal protuberance, makes this a distinctive species, with the most extreme head ornamentation that we have seen in *Genaemirum* (or any ichneumonid). The European *Auritus elephas* (Brauns) has genae expanded in a similar, but less extreme, fashion; however, we have only seen illustrations of this species (Tereshkin 2004). The colour pattern (yellow metasoma and infuscate wings) is also distinctive compared to *Genaemirum doryalidis*, which is the closest congener in its head ornamentation.

##### Etymology.

The name is derived from the genus name for warthogs, after the resemblance of the head to that of a warthog. Noun in apposition.

##### Description.

FEMALE. *Color.* Head black with face and large spot on vertex creamy-white, with narrow black border to clypeus and gena; mesosoma black, with creamy-white markings: anterior margin of pronotum (except medially), median 2/3 of mesopleuron, scutellum and large spot on posterior half of metapleuron; metasoma with first segment black, remainder dull yellow, paler posteriorly; flagellum uniformly black; legs black except hind tibia dull reddish brown, fore tibia brown on inner edge; wings infuscate, venation black, pterostigma pale brown; setae mostly pale, inconspicuous, but many setae on 6^th^ and 7^th^ tergites black.

*Head.* Mandible rather stout, slightly concave on outer aspect, mid-longitudinally punctate, teeth smoother with upper tooth about 2.5× as long as lower tooth; malar space 1.2× as long as mandible basal width; lower gena massively expanded as an elongate protuberance, apically rounded, concave and rugose on outer aspect with triangular projection on lower edge; in frontal view, genae ventrally diverging so that maximum breadth is 1.1× head width at widest point of eyes; ventral portion of occipital carina difficult to trace, one branch meets hypostomal carina far from base of mandible, another branch turned abruptly anterior then deflected to mandible base, delimiting strongly emarginate area on lower vertex; hypostomal carina distinctly expanded at apparent junction with occipital carina into a large, blunt, roughly equilateral triangle; clypeus about 0.9× as wide as high (measured at maximum height of projections from tentorial pits and width at tentorial pits), in profile slightly concave, not differentiated from face, ventral margin with elongate lateral lobes, face and clypeus coriaceous with punctures denser on clypeus, separated by 1.5–2× puncture width; upper face with conical projection between antennal sockets; lower frons quite smooth, flat and steeply elevated above toruli, with some rounded rugae forming a V-shaped slightly raised area medially, separated from upper frons by a transverse carina extended laterally into sharply pointed projections; upper frons coarsely rugose, rugose-striate around ocelli; vertex coriaceous with sparse punctures; temple distinctly narrowed behind eye; occipital carina raised, distinctly lamellar ventrally; ocellar triangle wider than long, ocelli rather small, POL 1.25, OOL 1.7; antenna stout, widened and slightly flattened medially, thinner apically, with 29 flagellomeres, first flagellomere 1.7× as long as apically wide, second 1.0× as long as wide, then widening towards middle with 8^th^ flagellomere 0.7× as long as wide.

*Mesosoma.* Pronotum ventrally finely and densely punctate fading dorsally to sparsely striate, pronotum collar rugose-striate, epomia strong; mesopleuron bulging, strongly convex, mostly coriaceous, smooth and shining posteriorly, speculum barely differentiated, epicnemium finely and densely punctate, otherwise mesopleuron scarcely punctate, posterior suture deeply crenulate, epicnemial carina nearly reaching pronotal margin below subtegular ridge, dorsally fading into striations, area below strongly rounded, expanded subtegular ridge rugose-striate; metapleuron coriaceous with large punctures, denser dorsally, crenulate along pleural carina; mesoscutum evenly rounded, mesoscutum and scutellum (mesoscutellum) coriaceous with very fine, sparse punctures, notaulus narrow, deep, distinct to anterior third, scuto-scutellar groove quite smooth, deep and narrow, scutellar margin overhanging; scutellum with lateral carina to about 2/3 of length, quite flat in profile; propodeum typical of genus, gently and evenly rounded in profile with median areas fused, carination distinct, enclosing elongate central area that is 2.2× wider posteriorly than anteriorly, densely punctate with punctation obsolescent mid-basally, coriaceous anteriorly and over entire area basalis+superomedia. *Legs.* Fore and mid tibiae with short and rather dense bristles along anterior faces, bristle sockets large and cupular.

*Metasoma.* Petiole with lateral and submedian dorsal carinae strong, abruptly enlarged into second tergite from level of spiracle, petiole coriaceous dorsally, more weakly sculptured on apex of postpetiole where sparsely punctate and faintly striate medially, tergite laterally strongly rugose; remainder of metasoma weakly coriaceous, more shining posteriorly, second tergite sparsely punctate laterally, medially longitudinally strigose; following tergites unevenly and sparsely punctate except tergite 2 medially and tergite 3 antero-medially longitudinally strigose, 4^th^ tergite with short area of median, anterior striae; gastrocoelus deep, thyridia separated by 1.7× their own width; 6^th^ and 7^th^ tergites with strong setae; ovipositor sheath slightly protruding beyond metasomal apex.

MALE. Unknown

##### Biology.

Unknown, but see discussion on biology under the genus treatment.

##### Distribution.

Tanzania.

#### Genaemirum fumosum

Taxon classificationAnimaliaHymenopteraIchneumonidae

Broad, Rousse & van Noort sp. n.

http://zoobank.org/75AB3798-E301-4AF0-BDC3-E5562E6F2A8D

Fig. 4

##### Type material.

**HOLOTYPE** ♀: [SOUTH AFRICA] ‘NISSV. Nelspruit 4/2/72 E. de Villiers’, ‘Genaemirum sp. ♀ det. T. Huddleston, 1972’ (BMNH).

##### Diagnosis.

*Female.* Body length 16 mm, fore wing length 11mm. Dark red mesosoma with creamy-white subtegular ridge and black propodeum; face, clypeus, lower 2/3 of vertex, spot on anterior edge of pronotum, all creamy-white; legs dark reddish brown to black except for dull yellow hind femur and tibia; metasoma dull yellow beyond blacl first segment; wings strongly infumate; antenna with 27 flagellomeres; lower gena slightly produced laterally as distinct ‘corners’ to the face in frontal view, gena flattened and produced posteriorly; hypostomal carina raised; clypeus flat, corners slightly produced so medially concave, with pointed tooth centrally; lower face densely punctate, with blunt projection between antennal sockets; upper frons rugose; scrobe smooth, impunctate; scrobe dorsally demarcated by raised, arched lamella, medially incised; mesosoma largely punctate, except mesoscutum, smooth and shining; metasoma weakly coriaceous, with sparse but distinct punctures, tergites 2-3 medially strigose, 4^th^ tergite strigose antero-medially, postpetiole medially striate. *Male.* Unknown.

##### Differential diagnosis.

Amongst *Genaemirum* species with a bisinuate, raised lamella above the antennal scrobes, *Genaemirum fumosum* sp. n. can be distinguished from *Genaemirum phagocossorum* sp. n. and *Genaemirum varianum* on colour pattern and by the shape of the gena; unlike *Genaemirum phagocossorum* sp. n. the metasoma and hind legs are largely dull yellow, as in *Genaemirum varianum*, although *Genaemirum fumosum* sp. n. differs from the latter in the more restricted pale markings on the mesosoma and in the more strongly infuscate wings, as well as the much more weakly produced gena. It shares with *Genaemirum phacochoerus* the overall colour pattern, including the infuscate wings, but differs markedly in head structure.

##### Etymology.

The name is derived from the Latin for "smoky", in reference to the infuscate wings. Noun in apposition.

##### Description.

FEMALE. *Color.* Head reddish brown with face and ventral 2/3 of vertex creamy-white, with narrow red border to clypeus; mesosoma dark reddish brown, with creamy-white subtegular ridge and small spot at ventral end of epomia, tegula, carinae at edge of scuto-scutellar groove, metascutellum, propodeum and metapleuron black; metasoma with first segment black, remainder dull yellow; flagellum uniformly black; legs with mid and hind coxae proximally black, fore and mid legs dark brown to black with fore tibia pale brown on inner side, mid tibia brown on inner side, hind leg with trochanter and trochantellus dark reddish brown, femur and tibia dull yellow, tarsus black; wings infuscate, venation dark brown (apically) to black, pterostigma black; setae mostly pale.

*Head.* Mandible rather stout, slightly concave on outer aspect, mid-longitudinally punctate, teeth smoother with upper tooth about twice as long as lower tooth; malar space 0.45× as long as mandible basal width; lower gena flattened and triangularly produced posteriorly, forming a partial genal bridge across the hypostoma, anteriorly, gena produced as a low, pointed projection; occipital carina ventrally meeting hypostomal carina distant from mandible base by 1.25× basal width of mandible; hypostomal carina raised, lamelliform; clypeus 2.6× as wide as medially high, flat, not differentiated from face, ventral margin with weakly produced lateral lobes and strong, pointed, median tooth, face and clypeus shining, densely punctate, especially medially, with blunt projection between antennal sockets, face 3.9× as wide as high; lower frons entirely smooth, flat and steeply elevated above toruli, separated from upper frons by a smoothly curved lamella, raised laterally, incised medially; upper frons coarsely rugose-striate, including between ocelli, some punctures between lateral ocelli; vertex smooth, very faintly coriaceous, with very inconspicuous, sparse punctures; temple about as wide as eye, roundly narrowed behind eyes; occipital carina sharp, lamellate ventrally; ocellar triangle wider than long, ocelli rather small, POL 1.3, OOL 1.2; antenna stout, widened medially, thinner apically, with 27 flagellomeres, first flagellomere 1.3× as long as apically wide, second 1.0× as long as wide, then widening towards middle with 8^th^ flagellomere 0.7× as long as wide.

*Mesosoma.* Pronotum densely punctate, shining with some rugosity posteriorly, ventrally, pronotum collar matt, epomia strong until just above transverse groove; mesopleuron a little bulging, mostly shining, densely punctate, speculum differentiated, unsculptured, epicnemium with finer, denser punctation, posterior suture deeply crenulate, epicnemial carina fading away distant from mid-height of pronotal margin, subtegular ridge rounded, expanded; metapleuron shining, densely punctate although punctation weaker centrally, short crenulations along pleural carina; mesoscutum evenly rounded, mesoscutum and scutellum (mesoscutellum) finely coriaceous, mesoscutum impunctate, scutellum with scattered punctures, notaulus narrow, distinct to anterior third, scuto-scutellar groove quite smooth, deep and narrow, scutellar margin overhanging; scutellum with lateral carina not extending beyond scuto-scutellar groove; propodeum typical of genus, gently and evenly rounded in profile with median areas fused, carination distinct except medially, enclosing elongate central area that is 1.7× wider posteriorly than anteriorly, shining and densely punctate with punctation obsolescent mid-basally, punctation very dense bordering pleural carina; *Legs.* Fore and mid tibiae with short and rather dense bristles along anterior faces, bristle sockets large and cupular.

*Metasoma.* Petiole with lateral and submedian dorsal carinae strong, abruptly enlarged into second tergite from level of spiracle, shining, postpetiole striate, punctate posteriorly, tergite laterally rugose-punctate; remainder of metasoma weakly coriaceous with distinct punctures, especially laterally on 2^nd^ and 3^rd^ tergites, medially longitudinally strigose; following tergites unevenly and sparsely punctate except tergite 4 antero-medially longitudinally strigose; gastrocoelus deep, thyridia separated by 2× their own width; 6^th^ and 7^th^ tergites with strong setae; ovipositor sheath slightly protruding beyond metasomal apex.

**Figure 4.**
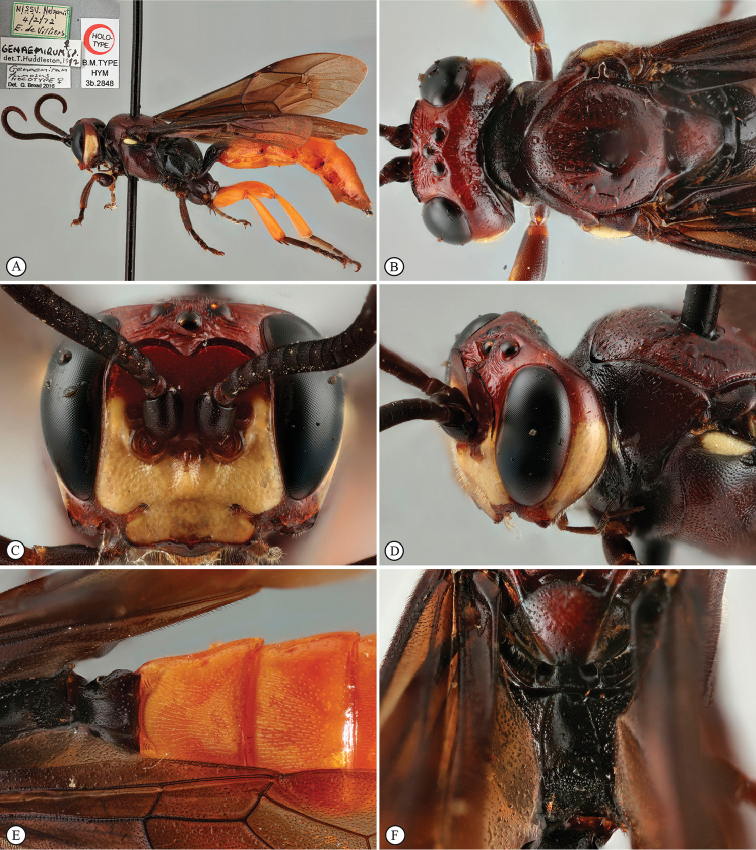
*Genaemirum fumosum* sp. n. Holotype female. **A** habitus lateral view (inset: data labels) **B** head, mesosoma, dorsal view **C** head anterior view **D** head, mesosoma anterior-lateral view **E** metasomal tergites 1-4 dorsal view **F** propodeum, dorsal view.

MALE. Unknown

##### Biology.

Unknown, but see discussion on biology under the genus treatment.

##### Distribution.

South Africa.

#### Genaemirum doryalidis

Taxon classificationAnimaliaHymenopteraIchneumonidae

Heinrich, 1967

Fig. 5

##### Material examined.

**Holotype** ♀: Type [red bordered circular label], Holotype [red label], KENYA, Kenya Forest Department, K. Elburgon, L0.704, ex *Dovyalis abyssinica*, Pres by Com Inst Ent BM 1965-3, C.I.E. 251, B.M. TYPE HYM. 3B.2120, *Genaemirum doryalidis* det. G. Heinrich, Name changed to *dovyalidis* G.H.H. i.l. to G.J.K., Imaged WaspWeb LAS 4.4 SAMC 2014 [yellow label] (BMNH).

##### Diagnosis.

*Female.* Body length 14 mm. Black with numerous white markings on head and mesosoma, fore leg orange from femur, and tergites 2–7 apically reddish; flagellum broken; lower gena extraordinarily expanded into flat, spade-shaped protuberance; clypeus strongly enlarged, considerably longer than face, widened and bent upward toward apex, concealing mandible and labrum in frontal view; frons rugose with two horn-like protuberances pointing upward; basal tergites longitudinally striate medially. *Male.* Unknown.

**Figure 5.**
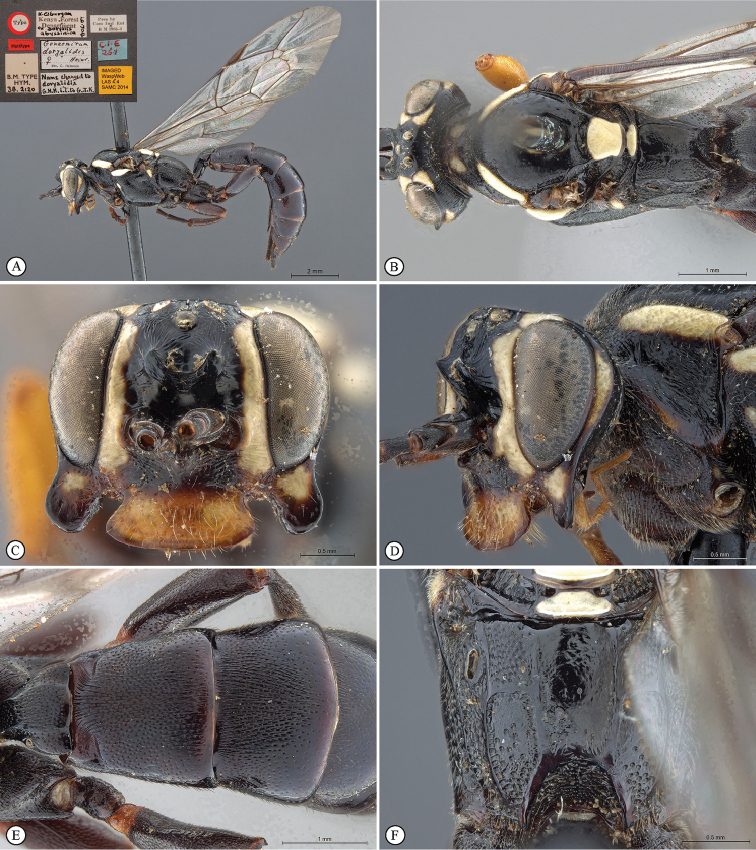
*Genaemirum doryalidis* Heinrich. Holotype female. **A** habitus lateral view (inset: data labels) **B** head, mesosoma, dorsal view **C** head anterior view **D** head, mesosoma anterior-lateral view **E** metasomal tergites 1-4 dorsal view **F** propodeum, dorsal view.

##### Biology.

Associated with *Dovyalis abyssinica* Warb. (Malpighiales: Salicaceae). See discussion on biology under the genus treatment.

##### Distribution.

Kenya.

#### Genaemirum mesoleucum

Taxon classificationAnimaliaHymenopteraIchneumonidae

Heinrich, 1936

Fig. 6

##### Material examined.

**Holotype** ♀: Typus [red label], KENYA Camp III de l’Elgon, Zone des Bruyèrès, est 3.500 m, Museum de Paris, Mission de l’Omo, C. Arambourg, P.A. Chappuis & R. Jeannel, 1932-33, *Genaemirum mesoleucum* ♀det. G. Heinrich, Imaged WaspWeb LAS 4.4 SAMC 2014 [yellow label] (MNHN).

##### Diagnosis.

*Female.* Body length 14 mm. Black, with dorsal white markings on head and mesosoma; flagellum with a whitish median ring; antenna with 33 flagellomeres; hypostomal carina extended onto malar space, expanded into a backward curved blunt triangle over lower gena; clypeus transverse, apical margin subtruncate; lower frons smooth, separated from upper frons by a transverse acutely M-shaped carina; upper frons rugose; ventral junction of occipital and hypostomal carinae moderately expanded, triangular; tergites 1–4 longitudinally striate medially. *Male.* Unknown.

**Figure 6.**
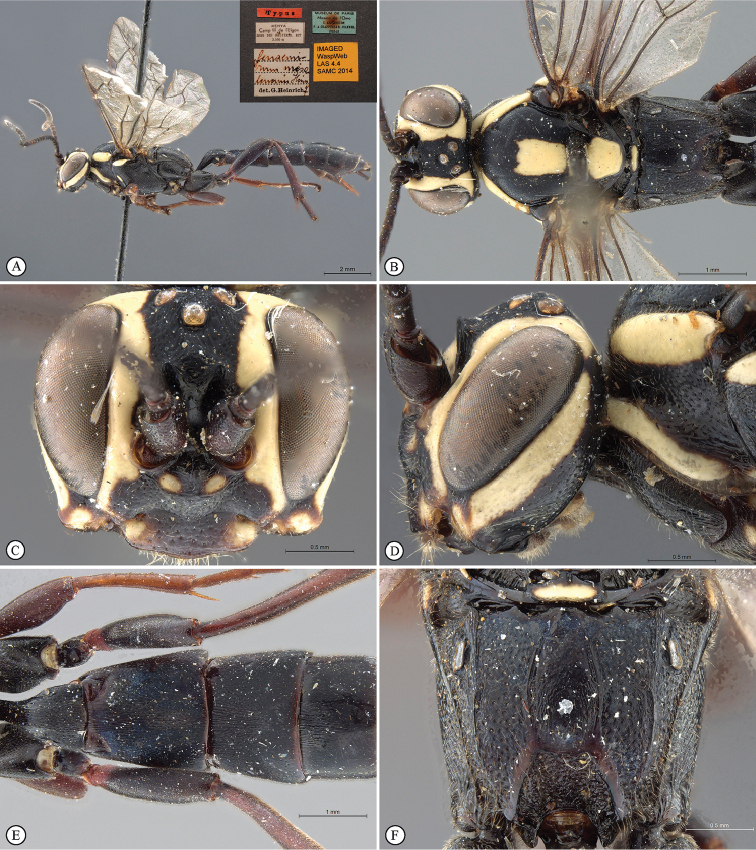
*Genaemirum mesoleucum* Heinrich. Holotype female. **A** habitus lateral view (inset: data labels) **B** head, mesosoma, dorsal view **C** head anterior view **D** head, pronotum lateral view **E** metasomal tergites 1-4 dorsal view **F** propodeum, dorsal view.

##### Biology.

Unknown, but see discussion on biology under the genus treatment.

##### Distribution.

Kenya.

#### Genaemirum rhinoceros

Taxon classificationAnimaliaHymenopteraIchneumonidae

Heinrich, 1967

Fig. 7

##### Material examined.

**Holotype** ♀: Holotype [red label] UGANDA, Zika Forest, Mengo, xi.18 ’63, G.A. Lancaster, Typus Nr. Hym. 436 Zoologische Staatssammlung München [red label], Zoologische Staatssammlung München, Type-No.: ZSM-Hym-00254 [pink label], *Genaemirum rhinoceros* det G. Heinrich, Imaged WaspWeb LAS 4.4 SAMC 2015 [yellow label] (ZSMC).

##### Diagnosis.

*Female.* Body length 15 mm. Head and mesosoma bright red interspersed with numerous large yellow markings; metasoma and legs yellowish-orange with coxae largely white; flagellum darker with a white median ring; antenna with 35 flagellomeres; hypostomal carina extended onto malar space, expanded into a backward curved blunt triangle over lower gena; clypeus lenticular, without differentiated ventro-lateral corners; face with a mid-longitudinal, horn-like, projecting lamella; lower frons smooth and short, separated from upper frons by a transverse M-shaped carina; upper frons with a differentiated rugose, triangular and shield-shaped surface; ventral junction of occipital and hypostomal carinae moderately expanded, triangular; tergites almost entirely punctate, without longitudinal sculpture except between gastrocoeli. *Male.* Unknown.

**Figure 7.**
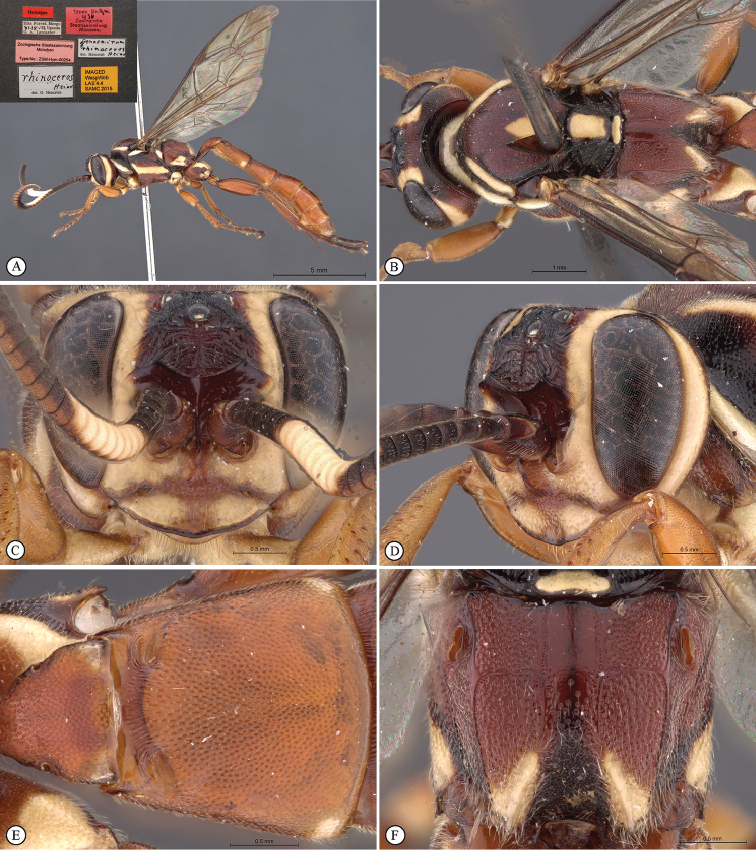
*Genaemirum rhinoceros* Heinrich. Holotype female. **A** habitus lateral view (inset: data labels) **B** head, mesosoma, dorsal view **C** head anterior view **D** head, pronotum anterior-lateral view **E** metasomal tergites 1-2 dorsal view **F** propodeum, dorsal view.

##### Biology.

Unknown, but see discussion on biology under the genus treatment.

##### Distribution.

Uganda.

#### Genaemirum varianum

Taxon classificationAnimaliaHymenopteraIchneumonidae

(Tosquinet, 1896)

Figs 8 , 9

Amblyteles varianus Tosquinet, 1896: 97

##### Material examined.

**Holotype** ♂: [South Africa] Type [red label], typus [red bordered label], Capland, Krebs., Zool. Mus. Berlin, 9213, ♂ *Amblyteles varianus* Tosquinet, Imaged WaspWeb LAS 4.4 SAMC 2016 [yellow label] (ZMHB). **Paratype** (Allotype)♀: [South Africa] Allotytpe [red label] Capland, Krebs, Zool. Mus. Berlin, 9213, ♀ *Amblyteles varianus* det. G. Heinrich, Imaged WaspWeb LAS 4.4 SAMC 2016 [yellow label] (ZMHB).

##### Diagnosis.

*Female.* Head and mesosoma red with white markings, mesosoma ventrally blackish; legs yellow with coxae red; antenna short with 26 flagellomeres; lower gena produced laterally in a blunt triangle curved backwards; ventral junction of occipital and hypostomal carinae sharply expanded, triangular; clypeus transverse, about rectangular with ventral margin subtruncate, with a blunt median tooth; lower frons smooth, separated from upper frons by a transverse bisinuate carina; upper frons and vertex multi-directionally striate; mesosoma punctate; metasoma lacking in the type (and only known) female. *Male.* Body length 15 mm.Head and mesosoma black with numerous white markings, except for mesoscutum red and centrally white marked; metasoma yellow with tergite 1 black; legs yellow, basally and apically black, with white markings on fore and mid coxae; lower gena without expansion and frons without transverse carina, but ventral junction of carinae similarly pointed.

**Figure 8.**
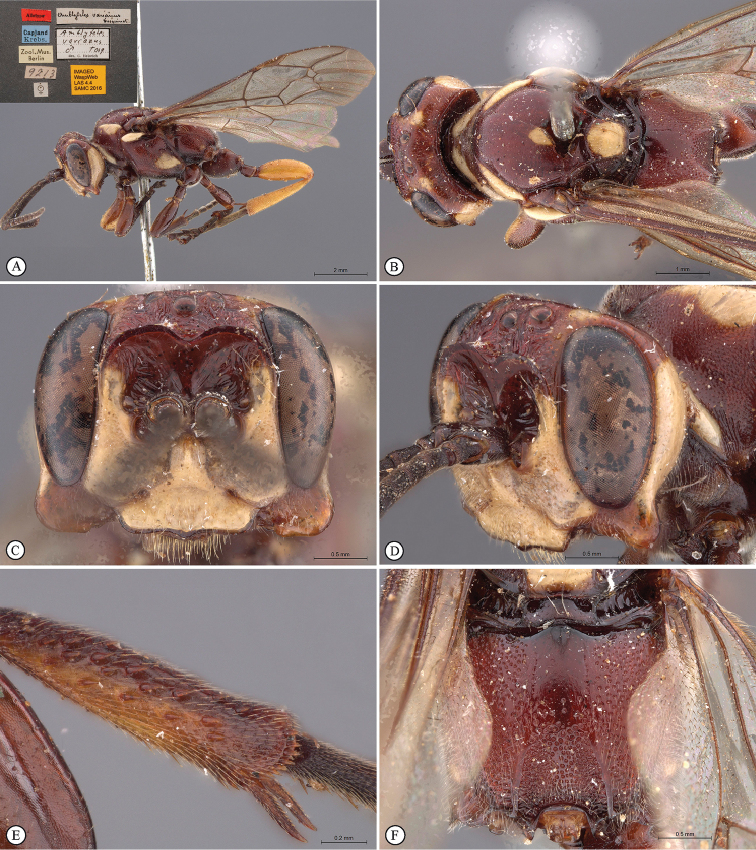
*Genaemirum varianum* (Tosquinet). Paratype female. **A** habitus lateral view (inset: data labels) **B** head, mesosoma, dorsal view **C** head anterior view **D** head, pronotum anterior-lateral view **E** fore-tibial armature **F** propodeum, dorsal view.

**Figure 9.**
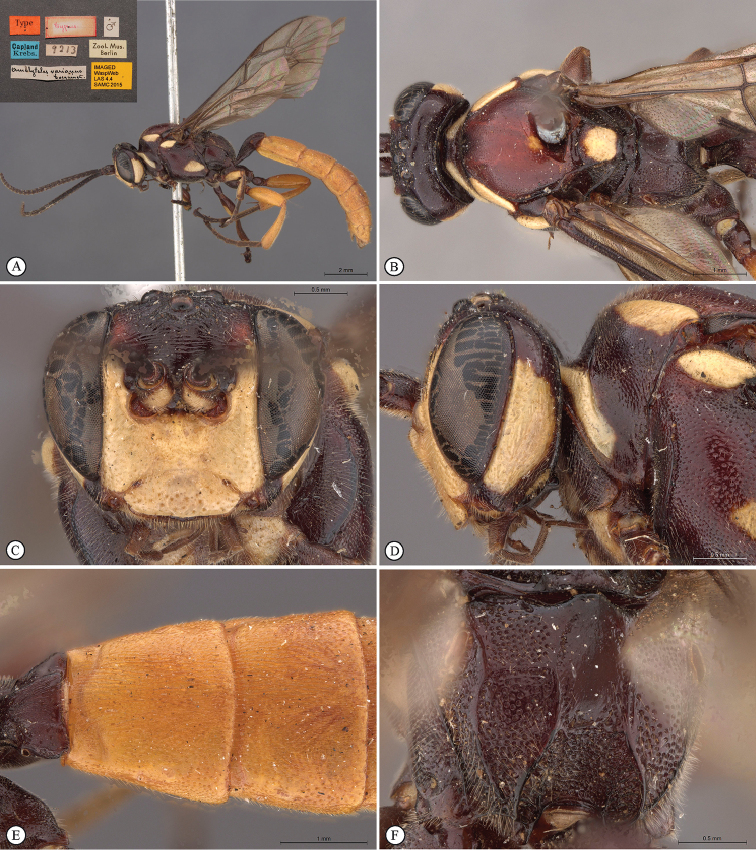
*Genaemirum varianum* (Tosquinet). Holotype male. **A** habitus lateral view (inset: data labels) **B** head, mesosoma, dorsal view **C** head anterior view **D** head, mesosoma anterior-lateral view **E** metasomal tergites 1-4 dorsal view **F** propodeum, dorsal view.

##### Biology.

Unknown, but see discussion on biology under the genus treatment.

##### Distribution.

South Africa.

#### Genaemirum vulcanicola

Taxon classificationAnimaliaHymenopteraIchneumonidae

Heinrich, 1967

Fig. 10

##### Material examined.

**Holotype** ♀: Holotype [red label] [TANZANIA] TANGANYIKA, Rungwe Mts., 2600 m, 12.XI.62, Typus Nr. Hym. 435 Zoologische Staatssammlung München [faded red label], Zoologische Staatssammlung München, Type-No.: ZSM-Hym-00253 [pink label], *Genaemirum vulcanicola* ♀ det. G. Heinrich, Imaged WaspWeb LAS 4.4 SAMC 2016 [yellow label] (ZSMC). 1♀, ‘Meru’, van Someren, VII.1943, V.G.L. van Someren collection, Brit. Mus. 1959-468 [there are towns called Meru in Kenya and Tanzania and van Someren’s collection is recorded only as being from East Africa, although Tanzania is perhaps more likely, as an ex-German colony] (BMNH).

##### Other material.

A female from South Africa (Natal: Kloof, 1500ft, VIII.1926, R.E. Turner) in BMNH is smaller and paler red than other specimens and has more abundant yellow markings, but is structurally very similar to the holotype and non-type female examined. We tentatively refer this specimen to *Genaemirum vulcanicola*.

##### Diagnosis.

*Female.* Body length 14 mm. Bright red overall with numerous yellow markings; flagellum darker with a white median ring; antenna with 37 flagellomeres; lower gena not expanded; clypeus transverse, about rectangular with ventral margin subtruncate; lower frons rugose, with two acute horn-like protuberances pointing upwards dorsally; ventral junction of occipital and hypostomal carinae expanded, pointed; tergites 1–4 longitudinally striate medially. *Male.* Unknown.

**Figure 10.**
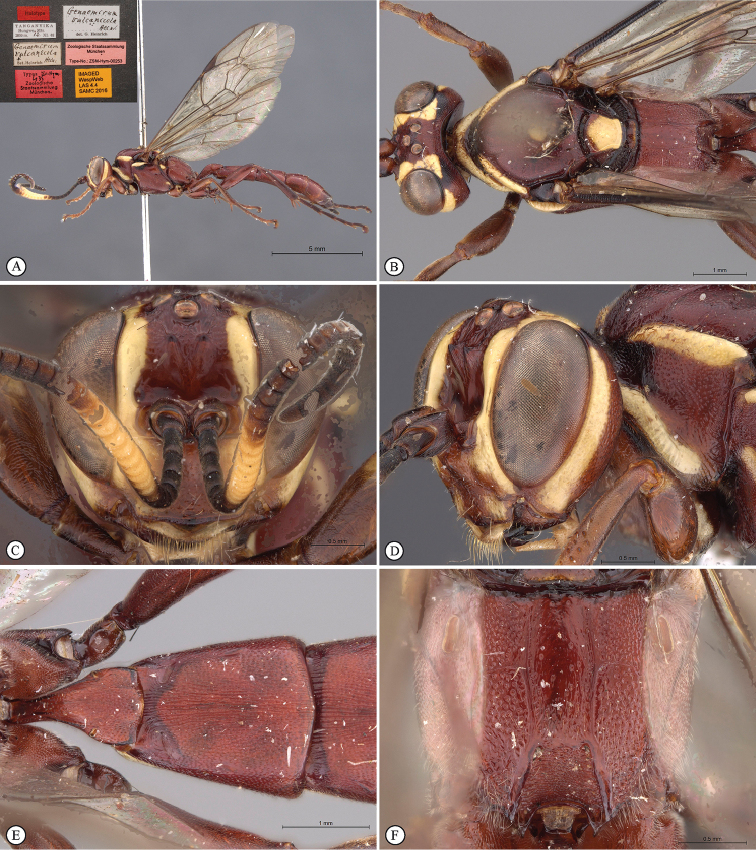
*Genaemirum vulcanicola* Heinrich. Holotype female. **A** habitus lateral view (inset: data labels) **B** head, mesosoma, dorsal view **C** head anterior view **D** head, mesosoma anterior-lateral view **E** metasomal tergites 1-4 dorsal view **F** propodeum, dorsal view.

##### Biology.

Unknown, but see discussion on biology under the genus treatment.

##### Distribution.

South Africa, Tanzania.

## Supplementary Material

XML Treatment for Genaemirum

XML Treatment for Genaemirum phagocossorum

XML Treatment for Genaemirum phacochoerus

XML Treatment for Genaemirum fumosum

XML Treatment for Genaemirum doryalidis

XML Treatment for Genaemirum mesoleucum

XML Treatment for Genaemirum rhinoceros

XML Treatment for Genaemirum varianum

XML Treatment for Genaemirum vulcanicola
